# Sleep Quality Among People with Type 2 Diabetes Mellitus During COVID-19 Pandemic: Evidence from Qatar’s National Diabetes Center

**DOI:** 10.2147/DMSO.S421878

**Published:** 2023-09-14

**Authors:** Yasamin Abdu, Sarah Naja, Mohamed Izham Mohamed Ibrahim, Mariam Abdou, Romaisa Ahmed, Salma Elhag, Ahmed O Saleh, Mohamed Yassin, Iheb Bougmiza

**Affiliations:** 1 Community Medicine Department, Hamad Medical Corporation, Doha, Qatar; 2 College of Pharmacy, QU Health, Qatar University, Doha, Qatar; 3 University of Elimam Almahadi, Khartoum, Sudan; 4 Wad Medani College of Medical Sciences and Technology, Jazeera, Sudan; 5 Department of Internal Medicine, Unity Hospital, Rochester, NY, USA; 6 Hematology Department, Hamad Medical Corporation, Doha, Qatar; 7 Community Medicine Department, Primary Health Care Corporation, Doha, Qatar; 8 Community Medicine Department, College of Medicine, Sousse University, Sousse, Tunisia

**Keywords:** poor sleep quality, sleep disturbance, T2DM, COVID-19, depression

## Abstract

**Purpose:**

Sleep disturbance is suspected to increase during the COVID-19 pandemic, and people with type 2 DM are known to have a higher risk of sleep disturbance. We aimed to determine the prevalence and determinants of sleep disturbance through Pittsburgh Sleep Quality Index (PSQI) during the COVID-19 pandemic.

**Patients and Methods:**

We randomly selected two hundred eighty-eight people with T2DM from the outpatient clinics of the National Diabetes Centre in Qatar. We used Chi-square, Mann–Whitney, Spearman, and Point Biserial correlation tests to examine the association between sleep quality and the independent variables. Finally, we conducted multiple logistics regression to identify the predictors of poor sleep quality and set the alpha level at 0.05.

**Results:**

In our sample, the mean age (±SD) was 51.4 (± 9.5) years, and 64.3% of the study participants were males. The median (IQR) duration of diabetes was 10 (11) years. Additionally, 6.3% of the participants were on insulin. The median HbA1c was 7.6% (2.4). Three in ten patients reported poor sleep quality (PSQI>5); (n=103; 35.8%). Poor sleep quality was statistically associated with young age, previous history of sleep disturbance, prior diagnosis of sleep disorders, high depression score, and high perceived stress score. After adjusting for confounders, only high depressive symptoms score and previous history of sleep disorder were significant predictors of poor sleep quality (p < 0.001), with adjusted odd ratios of (aOR = 1.421; 95% CI: 1.242–1.625) and (aOR = 3.208; 95% CI: 1.574–6.537), respectively.

**Conclusion:**

The prevalence of poor sleep quality among people with T2DM during the COVID-19 pandemic is high. Physicians must screen for depression, stress, and previous history of sleep disorder to tackle poor sleep among T2DM.

## Introduction

Part of the crucial human well-being pillars is good sleep hygiene, which includes adequate duration, appropriate timing, good quality, and lack of sleep disturbances.^1^,^2^

Sleep disorder prevalence is around 20% in the general population (insomnia 6–15%, restless leg syndrome affects 6%, sleep paralysis affects about 6%, and sleep apnea affects around 2–4%). However, the prevalence of this disorder is nearly double among people with type 2 Diabetes Mellitus (T2DM).^3–5^ Where cumulative evidence from thirty-six studies revealed that the prevalence of sleep disorders among people with T2DM is 52%, specifically 69% of people with T2DM suffer from unspecified sleep apnea, and 60% have obstructive sleep apnea.^6^

Glycemic control is multifactorial; it is affected by low self-care activities,^7–10^ and psychosocial factors such as stress and depression.^11^,^12^ In addition, sleep disorders negatively impact the health of people with T2DM, increasing the risk of nocturia, peripheral neuropathy, fluctuations in blood glucose levels during the night, and depression.^13^ Specifically, inappropriate sleep time interval (less than 6 hours and more than 9 hours), alterations of sleep duration, chronic sleep restriction, excessive sleep; sleep fragmentation; circadian rhythm disorders and disruption (ie, shift work); and obstructive sleep apnea (OSA) influence glycemic control and provoke complications.^14–16^

Sleep disturbance, sleep fragmentation, and hypoxia increase sympathetic activity decrease insulin sensitivity and thus increase blood glucose levels.^17^ Moreover, the frequent arousal episodes caused by respiratory disturbance and the consequent sleep loss may enhance the development of metabolic disorders.^18^

Sleep disturbance is suspected to increase in stressful conditions such as the spread of the lethal viral infection and lockdown policies, including confinement at home, working from home, and distance learning that increases fear and disrupts the sleep-wake cycle, circadian and homeostatic factors.^19^

Diabetes is one of the critical non-communicable diseases that affect the population in Qatar. The prevalence of Type 2 Diabetes mellitus among aged 18–64 in 2021 was 10.4%.^20^

Assessment of sleep quality, sleep disorders, and sleep hygiene has a massive importance in people with T2DM. The latest American Diabetes Association 2023 guidelines^21^ recommended that assessment of sleep pattern and duration should be a part of the comprehensive medical evaluation in people with T2DM based on emerging evidence about the relationship between sleep quality and glycemic control. Before the pandemic, more than 50% of people withT2DM had excess daytime sleepiness and suffered from low sleep quality.

In Qatar, a study conducted in 2009^22^ showed that 60.1%of T2DM patients had excess daytime sleepiness; however, this study did not investigate the effect of sleep quality on disease control. Another recent study^23^ assessed sleep quality and its impact on glycemic management using data from Qatar Biobank; however, they studied only the sleep duration part and the daytime napping. To our knowledge, there is no study in Qatar conducted among diabetic patients during COVID-19 to assess the burden of poor sleep quality and the associated factors, as well as its potential impact on glycemic control, using a validated tool with high sensitivity and specificity (PSQI). Our study aimed to fill the gap to inform the policy makers about the prevalence and burden of poor sleep quality among diabetic patients during the pandemic, reflecting on the programs and policies needed at the tertiary health-care level.

## Materials and Methods

### Study Design and Recruitment

We conducted an analytical cross-sectional study. In addition, we surveyed patients attending the National Diabetes Centre in Hamad General Hospital from January 2021 and June 2022. The National Diabetes Centre is the main center for diabetes in Qatar,^24^ providing tertiary care through a multidisciplinary team staffed by endocrinologists, retinal specialists, podiatrists, nutritionists, education specialists, nurses, pharmacists, and technicians who all deliver diabetes care.

The National Diabetes Centre receives more than 120,000 visits and over 30,000 people with T2DM annually; it provides health care for the entire registered population, nationals, and expatriates. Specifically, people with T2DM who have outside glucose target and those eligible for T2DM complications screening receive care in this center.

We selected participants randomly from the attendance list using an automated random number generator, a simple random technique. Participants were interviewed after securing written consent. The target population included adult patients (18 years and above) diagnosed with T2DM and able to communicate in Arabic or English. We excluded pregnant women and patients with cognitive impairment that interfered with communication. A written informed consent was obtained from all the study participants. Ethical approval was obtained from the Institutional Review Board (IRB) of Hamad Medical Corporation (MRC-01-21-317).

### Sample Size Equation

We utilized the prevalence of poor sleep quality among people with type 2 DM in Qatar prior to the pandemic of 60%,^22^ with a 5% degree of precision and 95% confidence limits. Accordingly, the minimum sample size of 370 was calculated using the following formula:^25^

$${\mathrm{n}}={{ [\kern-0.15em[\left[{z21-{1 \over 2}{ ]\kern-0.15em] _x}px(1-p)} \right]}\over{d2}}$$

### Variables and Measurement Tools

Sleep quality is our dependent variable; it is theoretically defined as the person or individual’s self-satisfaction with all aspects of the sleep experience. Four domains contribute to sleep quality: wake after sleep onset, sleep efficiency, latency, and duration.^26^

We used the English and Arabic versions of the Pittsburgh Sleep Quality Index (PSQI) to assess sleep. It is a nineteen-item self-rated questionnaire to evaluate sleep quality over the previous month across seven component domains: subjective sleep quality, sleep latency, sleep duration, habitual sleep efficiency, sleep disturbances, use of sleep medication, and daytime dysfunction. Each component scored from zero to three; the higher the score, the more significant the sleep problem.

The PSQI demonstrated great criterion validity for the English and Arabic versions. A score of five and above indicated poor sleep quality with an acceptable sensitivity (89.6%) and specificity (86.5%). Furthermore, good internal consistency alpha=0.77.^27–29^

Regarding the independent variables, Personal interviews using the structured questionnaire were conducted with people with T2DM to collect information about sociodemographic and health-related characteristics (the independent variables) such as COVID-19 infection and vaccination status, health habits, comorbidities, diabetes complications, latest Hemoglobin A1C (HbA1C) in the previous 3 months, and lipid profile.

Perceived stress is one of the essential dependent variables that can influence sleep, and it is defined as the degree to which patients appraise situations in their lives as stressful. We utilized the English and Arabic versions of the self-administered Perceived Stress Scale (PSS-4) to assess the perceived stress level.^30^ The scale consisted of four items and showed acceptable internal validity (alpha=0.8). There is no established cutoff point for (PSS-4), but the higher the perceived stress level.

As for depressive symptoms assessment, we utilized the English and Arabic versions of the Patient Health Questionnaire (PHQ-9). It is a self-administered instrument that is shown to be a good screening tool with 88% sensitivity and 88% specificity for major depression when utilizing a cutoff of 10.^31^ In addition, the Arabic version of PHQ-9 showed acceptable reliability in a neighbouring country Saudi Arabia (alpha=0.857).^32^

### Analysis

We utilized software package SPSS version v29 (Armonk, NY: IBM Corp) for statistical analysis. Quantitative variables were described in the form of mean and standard deviation, while qualitative variables were presented in the form of frequency and percentage. Inferential statistics, ie, Mann–Whitney, Chi-Square, Spearman correlation, and Point Biserial correlation tests, were used to test the association between sleep quality and the independent variables. In addition, multiple logistic regression was carried out to identify the predictors of poor sleep quality among people with T2DM. The a priori alpha level was set at 0.05.

## Results

### Sample Size Realization

During the data collection period (January 2021 to June 2022), we approached three hundred seventy people with T2DM and two hundred eighty-eight participants completed the study, with a twenty-two percent non-response rate.

### Sociodemographic and Health-Related Characteristics of the Sample

The patients’ mean ± (SD) age was 51 ± (9.5) years. More than 50% of the sample were males and employed. The median (IQR) of the perceived stress score and patient health questionnaire nine score among people with T2DM was 8 (3) and 1 (3), respectively. The duration of T2DM ranged between one and forty-five years, with a median (IQR) of 10 (11). Less than 10% were on insulin (n=15; 6.3%), and one-third of the sample (n=65; 28.3%) were on combined insulin therapy and other antihyperglycemic agents. Most of our patients had blood glucose level above the target (HbA1c >7%) with desirable levels of LDL (3.36–4.11 mmol/L), HDL (>1 mmol/L), and total cholesterol (<5 mmol/L) 63%, 85.5%, 72.7%and 78.2%, respectively. Ten percent of the sample was diagnosed with sleep disturbance before COVID-19, as seen in Table 1.

**Table 1:** Sociodemographic and Health-Related Characteristics of Patients with T2DM at the National Diabetes Center (N=288)

	Frequency (n)	Percentage (%)
**I-Sociodemographic**		
**Gender**		
Male	184	(63.9)
Female	102	(35.7)
**Marital Status**		
Single	13	(4.6)
Divorced	9	(3.2)
Married	252	(88.4)
Widow	11	(3.9)
**Education level**		
ICan not read or write	14	(4.9)
Primary school	32	(11.3)
Secondary school	80	(28.2)
University and higher education	158	(55.6)
**Occupation**		
Unemployed	80	(28.8)
Job without night shift	157	(56.5)
A job with night shift	41	(14.7)
**Monthly family income**		
Up to 10,000 QAR	127	(49.8)
10,001–20,000 QAR	65	(25.5)
More than 20,000 QAR	63	(24.7)
**II-COVID-19 Exposure**		
**Previous infection with COVID-19**		
Yes	87	(30.6)
No	197	(69.4)
**Fully vaccinated againstCOVID-19**		
Yes	265	(93.3)
No	19	(6.7)
**III-Comorbidities**		
**Hypertension**		
Yes	151	(53.4)
No	132	(46.6)
**Diabetic complications (≥ one complication)**		
Yes	103	(36.4)
No	180	(63.6)
**IV-Behavioral**		
**Obesity**		
Underweight	20	(7.6)
Normal BMI	126	(47.9)
Overweight	64	(27.8)
Obese	44	(16.7)
**Smoking history**		
Yes	36	(12.6)
No	250	(87.4)
**Alcohol drinking**		
Yes	12	(4.2)
No	274	(95.8)
**V-Sleep history**		
**History of sleep disturbance**		
Yes	62	(21.9)
No	221	(78.1)
**Prior diagnoses of sleep disturbance**		
Yes	28	(9.9)
No	255	(90.1)

**Abbreviations**: n, frequency; %, percentage; SD, Standard Deviation.

### Prevalence of Poor Sleep Quality

Nearly one-third of the sample reported poor sleep quality (n=83; 35.8%), and Table 2 shows the distribution of the different components of the PSQI score. The median (IQR)age of those patients with poor sleep quality was 50 (14) and 53 (13) in patients with good sleep quality.

**Table 2:** Component of PSQI Score of Patients with T2DM at the National Diabetes Center

Component of PSQI	Median	IQR
Subjective sleep quality	0.00	1.00
Sleep latency	0.00	2.00
Sleep duration	1.00	2.00
Sleep efficiency	0.00	0.00
Sleep disturbance	1.00	0.00
Use of sleep medication	0.00	0.00
Daytime dysfunction	0.00	0.00

### Determinants of Poor Sleep Quality

Poor sleep quality was significantly associated with the participant’s age, perceived stress, and depression score; moreover, previous history of sleep disturbance and diagnosis were found to be significant determinants. However, no statistical association was found between poor sleep quality and other sociodemographic variables, as seen in Table 3.

**Table 3:** Determinants of Poor Sleep Quality of Patients with T2DM at the National Diabetes Center (N=288)

	Good Sleep Quality		Poor Sleep Quality		
**I-Categorical determinants**	n	(%)	n	(%)	p-value
**Gender**					
Male	116	(67.4)	57	(58.8)	0.154
Female	56	(32.6)	40	(41.2)	
**Marital status**					
Single	6	(3.5)	6	(6.3)	0.148
Married	155	(90.1)	81	(84.4)	
Widow	8	(4.7)	3	(3.1)	
Divorced	3	(1.7)	6	(6.3)	
**Educational level**					
Can not read or write	12	(7.0)	1	(1.0)	0.133
Primary school	20	(11.7)	9	(9.4)	
Secondary school	50	(29.2)	28	(29.2)	
Higher education	89	(52.0)	58	(60.4)	
**Employment status**					
Unemployed	50	(29.2)	28	(29.2)	0.990
Employed	121	(70.8)	68	(70.8)	
**Monthly family income (QAR)**					
Up to 10,000	83	(52.5)	36	(44.4)	0.494
10,001–20,000	39	(24.7)	23	(28.4)	
≥ 20,001	36	(22.8)	22	(27.2)	
**History of sleep disturbance**					
Yes	20	(11.7)	36	(37.9)	< 0.001
No	151	(88.3)	59	(62.1)	
**Prior diagnosis with sleep disorders**					
Yes	6	(3.6)	19	(20.0)	< 0.001
No	165	(96.5)	76	(80.0)	
**II-Continuous determinants**	Median (Δ IQR)		Median (Δ IQR)		
Age	53.0 (13.0)		50.0 (14.0)		0.004
Perceived psychological stress score	8.0 (4.0)		8.0 (2.0)		0.001
Depression score	1.0 (2.0)		3.0 (5.0)		< 0.001
Diabetes duration	10.0 (11.0)		9.0 (10.0)		0.496
Glycemic control (HbA1C)	7.7 (3.0)		7.4 (2.0)		0.129

**Abbreviations**: Δ IQR, Inter quartile range; n, frequency; %, percentage.

### Correlations

Perceived stress and depressive symptoms scores were positively correlated with sleep quality scores, as shown in Table 4.

**Table 4:** Correlation Between Sleep Quality Index, HbA1c, Perceived Psychological Stress Score, and Depression Score Among Patients with T2DM at the National Diabetes Center (N=288)

Spearman’s Rho		Last HbA1C	PSS4 Total Score	PHQ9 Total Score	PSQI Total Score
Last HbA1C	Correlation Coefficient	1	−0.099	−0.077	−0.093
	Sig. (2-tailed)		0.105	0.215	0.137
	N	270	267	260	256
PSS4 total score	Correlation Coefficient	−0.099	1	0.279**	0.231**
	Sig. (2-tailed)	0.105		<0.001	<0.001
	N	267	283	273	267
PHQ9 total score	Correlation Coefficient	−0.077	0.279**	1	0.498**
	Sig. (2-tailed)	0.215	<0.001		<0.001
	N	260	273	276	265
PSQI total score	Correlation Coefficient	−0.093	0.231**	0.498**	1
	Sig. (2-tailed)	0.137	<0.001	<0.001	
	N	256	267	265	271

**Notes**: **Correlation is significant at the 0.01 level (2-tailed).

**Abbreviations**: HbA1C, hemoglobin A1C; PSS4, Perceived Stress Scale 4; PHQ-9, Patient Health Questionnaire-9; PSQI, Pittsburgh Sleep Quality Index.

### Predictors

We ran binary logistic regression to determine the significant predictors for poor sleep quality (coded as 1). We included significant variables (ie, age, previous history of sleep disorders, depression score, and perceived psychological stress score) of the preliminary analysis of the model. The regression revealed depression as a significant predictor of poor sleep quality. Patients with higher depression scores were 1.4 times more likely to experience poor sleep quality (Adjusted OR = 1.421; 95% CI: 1.242–1.625) than patients with good sleep quality. Furthermore, patients with an earlier history of sleep disorder were 3.2 times more likely to experience poor sleep quality (Adjusted OR = 3.208; 95% CI: 1.574–6.537) than patients with good sleep quality. Age and higher stress scores failed to predict poor sleep quality (p>0.05), as seen in Table 5.

**Table 5:** Predictors of Poor Sleep Quality Among Patients with T2DM at the National Diabetes Center (N=288)

	PSS4 Total Score	95% CI for Adjusted OR		p-value
	Adjusted OR	Lower	Upper	
Age	1.009	0.975	1.044	0.608
PSS4 score	1.059	0.954	1.175	0.282
PHQ9 score	1.416	1.24	1.618	<0.001
Previous history of sleep disorder	3.208	1.574	6.537	<0.001
Constant	0.08			0.017

**Notes**: Variable(s) entered on step 1: age, PSS4 total score, PHQ9 total score, previous history of sleep disorder.

**Abbreviations**: PSS4, Perceived Stress Scale 4; PHQ-9, Patient Health Questionnaire-9; PSQI, Pittsburgh Sleep Quality Index.

## Discussion

This study investigated the sleep quality among people with T2DM visiting the National Diabetes Center in Qatar during the COVID-19 pandemic. Almost one-third of the participants (35.8%) demonstrated poor sleep quality. In addition, a significant association was found between poor sleep quality and a previous history of sleep disturbance, high perceived stress, and depressive symptoms.

Our study showed that the prevalence of sleep disturbance among people with diabetes during the pandemic is significantly higher than the prevalence reported among the general population during the pandemic in Qatar (5%).^33^

Few studies investigated sleep quality among people with diabetes during the pandemic. In Southern Brazil, social distancing, home confinement, practicing less physical activity, and irregular sleep times contributed to sleep disorders in three-quarters of a cohort of people with type 2 diabetes.^34^ Furthermore, half of the participants reported taking morning naps and 30% perceived worse sleep quality through Mini-Sleep Questionnaire (MSQ).^34^ Despite using different sleep assessment tools, they revealed a similar prevalence to our study. Possible explanations include concerns about the risk of contracting COVID-19 while having diabetes that can play an important role and fluctuating blood sugar levels with frequent hyperglycaemia and hypoglycaemia episodes during the night leading to insomnia and next-day fatigue.^34^

We found that age is a significant determinant for poor sleep quality, which could be explained by the fact that young adults with diabetes exhibit more stage two sleep and tend to have less deep sleep during the first half of the night.^35^ However, later this variable failed to predict sleep quality in the logistic model, indicating its confounding effect.

Considering gender, our analysis revealed that gender is not statistically related to poor sleep quality. Similar to another study implemented in southeast India.^36^

Regarding sleep duration, this clinical variable did not affect sleep quality among people with diabetes. Our finding is similar to a study conducted in the United States.^37^ However, in Ethiopia, they revealed that diabetes duration could be a significant determinant of poor sleep quality. Specifically, patients over 10 years were three times more likely to have poor sleep than individuals with a shorter duration of diabetes.^38^

Sleep disorders and depression are closely related. For example, in our study, high depressive symptom scores were associated with poor sleep quality. Moreover, a statistically significant positive correlation was found between them, similar to another survey done in the USA.^39^ In one study, 41% of patients experienced a sleep disorder before a major depressive disorder;^40^ also, other studies found that people with depression usually have difficulties falling asleep, frequent sleep fragmentations, and early morning get-up time,^38^,^41^ and this might be attributed to the fact that insomnia is one of the typical early symptoms of depression. Moreover, depressed patients usually present with physical symptoms such as fatigue and sleep disturbance.^42^

A high perceived stress score was significantly associated with poor sleep quality. Fear, anxiety, and stress about a novel disease and what could happen can be and can have a considerable impact on people withT2DM and can disrupt their psychosocial life. Other studies have also documented the association between psychological distress and poor sleep quality among people with T2DM.^43–45^

In our study, we did not find a statistically significant association between poor sleep quality and the control of HbA1C, in line with other studies.^46–48^ In contrast, two studies^49^,^50^ found a statistically significant association between sleep quality and HbA1C.

To our knowledge, this study is the first to investigate the prevalence of low sleep quality during the pandemic. We selected patients randomly (probability sampling) and from the main diabetes centre in Qatar to ensure the generalization of results. However, our study comes with limitations. First, we did not use diagnostic measures to assess sleep quality (lack of polysomnography and lack of actinography). Second, we utilized a cross-sectional design that compromised the temporality and causality of the association between the variables.

Despite these limitations, our results will significantly impact public health. Using fully adjusted models, we found that high depression scores and a history of sleep disorder were significant predictors of poor sleep quality among people with T2DM; thus, clinicians caring about those patients should screen for and treat depressive symptoms and poor sleep quality. Moreover, a psychologist should be involved in the routine care of those patients, which can help in tackling both depressive symptoms and sleep disturbance, which can improve the overall quality of life of people with T2DM.

## Conclusion

Our study showed that poor sleep quality is prevalent among people with type 2 during Covid 19; moreover, high depressive symptoms score and prior history of sleep disorders significantly predict poor sleep quality among those patients. Therefore, clinicians should screen for and treat sleep difficulties and depressive symptoms in people with type 2 diabetes.
